# Inhibitory effect of probiotic yeast *Saccharomyces cerevisiae* on biofilm formation and expression of α-hemolysin and enterotoxin A genes of *Staphylococcus aureus*

**Published:** 2019-06

**Authors:** Navid Saidi, Parviz Owlia, Seyed Mahmoud Amin Marashi, Horieh Saderi

**Affiliations:** 1 Department of Microbiology, School of Medicine, Shahed University, Tehran, Iran; 2 Molecular Microbiology Research Center (MMRC), Shahed University, Tehran, Iran; 3 Department of Microbiology, Qazvin University of Medical Sciences, Qazvin, Iran

**Keywords:** Biofilm, Exotoxins, Probiotic, *Saccharomyces cerevisiae*, *Staphylococcus aureus*

## Abstract

**Background and Objectives:**

*Staphylococcus aureus*, as an opportunistic pathogen, is the cause of a variety of diseases from mild skin infections to severe invasive infections and food poisoning. Increasing antibiotic resistance in *S. aureus* isolates has become a major threat to public health. The use of compounds produced by probiotics can be a solution to this problem. Thus, the purpose of this study was to investigate the effect of *Saccharomyces cerevisiae* on some virulence factors (biofilm, α-hemolysin, and enterotoxin A) of *S. aureus*.

**Materials and Methods:**

Supernatant and lysate extracts were prepared from *S. cerevisiae* S3 culture. Sub-MIC concentrations of both extracts were separately applied to *S. aureus* ATCC 29213 (methicillin-sensitive *S. aureus*; MSSA) and *S. aureus* ATCC 33591 (methicillin-resistant *S. aureus*; MRSA) strains. Biofilm formation of these strains was measured by microtiter plate assay and expression level of α-hemolysin and enterotoxin A genes (*hla* and *sea*, respectively) using real-time PCR technique.

**Results:**

The supernatant extract has reduced both biofilm formation and expression of *sea* and *hla* genes, while lysate extract had only anti-biofilm effects. The MRSA strain showed more susceptibility to yeast extracts than MSSA strain in all tests.

**Conclusion:**

The present study exhibited favorable antagonistic effects of *S. cerevisiae* S3, as a probiotic yeast, on MSSA and MRSA strains. Based on the findings of this study, the compounds produced by this yeast can be used to control *S. aureus* infections; however, further similar studies should be conducted to confirm the findings of the present study.

## INTRODUCTION

*Staphylococcus aureus* is an opportunistic pathogenic Gram-positive bacterium that causes a wide range of infections. Acute infections, such as septicemia, skin abscesses, and food poisoning, are mainly developed by secreting exoenzyme and toxins, but chronic infections occur due to biofilm formation (1, 2). *S. aureus* has several exotoxins, namely, hemolysins and enterotoxins. The α-hemolysin plays an important role in the pathogenesis of *S. aureus*. This toxin causes osmotic lysis and degradation with the expansion of pores across the lipid bilayer of cell membrane. Moreover, the α-hemolysin can cause tissue damage by altering cellular signaling pathways and inflammatory responses (3, 4). Staphylococcal enterotoxins (SEs) are potent gastrointestinal exotoxins, whose consumption develops staphylococcal food poisoning (5). *S. aureus* enterotoxins are among the important bacterial superantigens. These toxins produce large amounts of cytokines by stimulating a large number of T-cells (6). Among SEs, SEA is the most common source of staphylococcal food poisoning, and *sea* is the most prevalent isolated enterotoxin gene among *S. aureus* isolates (5, 7). Staphylococci are known as the leading cause of biofilm-related infections (8). *S. aureus* can attach and form biofilm on the host tissue and indwelling medical devices (1). The initial attachment of *S. aureus* is accomplished by microbial surface components recognizing adhesive matrix molecules (MSCRAMMs); and then, extracellular polymeric substances cause cell-to-cell junction and biofilm formation. The biofilm increases bacterial resistance to antibiotics and host defense mechanisms (8, 9).

The emergence of antibiotic-resistant strains of *S. aureus* is a major threat to public health. Therefore, it is highly recommended to conduct more studies to find new methods to prevent and control infections of this bacterium. Recently, probiotics have been considered as natural and healthy microorganisms against antibiotic-resistant and corrosive food microbes to replace antibiotics and chemical preservatives (10, 11). Many studies have been conducted on *S. cerevisiae*, a single-cell yeast, and its genetics and industrial applications. Many previous studies have shown that *Saccharomyces* strains can have antimicrobial and probiotic properties (12). Competing for food, reducing the pH of the environment, producing high-concentration ethanol, and secreting antimicrobial compounds are some of the most important antagonistic effects of yeasts (13).

The aim of this study was to examine the effects of *S. cerevisiae* on the biofilm formation, the hemolytic activity, and the expression of α-hemolysin and enterotoxin A genes (*hla* and *sea*, respectively) in 2 standard strains of methicillin sensitive and resistant *S. aureus*.

## MATERIALS AND METHODS

### Bacterial and yeast strains.

This study, conducted at the Molecular Microbiology Research Center (MMRC) of Shahed University, used a native strain of *S. cerevisiae* S3 with a probiotic property isolated in previous studies (14, 15). The yeast was kept at −70°C in Potato Dextrose Broth (PDB) (Ibresco, Iran) containing 20% glycerol. The bacterial strains of *S. aureus* ATCC 29213 (methicillin-sensitive *S. aureus*; MSSA) and *S. aureus* ATCC 33591 (methicillin-resistant *S. aureus*; MRSA) were cultured in nutrient agar medium and kept at −70°C in nutrient broth containing 15% glycerol.

### *Staphylococcus aureus* culture condition.

Four colonies of *S. cerevisiae* S3 yeast were transferred to 20 mL of PDB medium and incubated at 30°C for 16 hours (late log phase), with shaking rate of 230 rpm. This overnight culture was used to inoculate 1000 mL of PDB medium and was divided into four 250 mL flasks. The flasks were incubated at 30°C for 24 hours with 120 rpm and centrifuged at 3000 g for 10 minutes to collect supernatant and cell pellet (16).

### Preparation of *S. cerevisiae* supernatant and lysate extracts.

The supernatant extract of *S. cerevisiae* S3 was obtained in accordance with the method previously described by Krasowska et al. with a slight modification (16). At first, the supernatant was passed through a 0.22 μm filter and then extracted with ethyl acetate over 3 hours. Therefore, the supernatant was mixed in a ratio of 5 to 1 with ethyl acetate. The ethyl acetate was exchanged every half hour and then removed by rotary evaporator (Hei-VAP Advantage ML, Heidolph, Germany) to obtain dry substance. Typically, about 45–50 mg of dry substance was obtained from 1 liter of *S. cerevisiae* S3 culture. By adding a suitable amount of methanol to the dry substance, the extract was prepared at a concentration of 327.68 mg/mL, which was stored as supernatant extract stock. The *S. cerevisiae* S3 lysate was prepared using the sonicator (Q125 Sonicator, Qsonica, USA). Therefore, the yeast pellet obtained in the previous step was washed once with distilled water and dissolved again in distilled water. The resulting suspension was lysed by the sonicator at 4°C. Each sonication cycle was performed in 20 minutes, with 50-sec pulse on and 10-sec pulse off program and 100% amplitude. The lysate was centrifuged at 15 000 rpm at 4°C for 30 minutes and then passed through a filter of 0.45 μm (17). The water was removed from the lysate by the rotary evaporator, and 180–200 mg of lysate dry substance was harvested. By adding the appropriate amount of double-distilled water to the dry lysate, the lysate extract stock was prepared at a concentration of 163.84 mg/mL.

### Minimum inhibitory concentration (MIC) and minimum bactericidal concentration (MBC) of the extracts.

To determine the MIC, broth microdilution method was used in TSB medium (Merck, Germany) (18). By adding appropriate amounts of TSB medium to the yeast supernatant and lysate extracts, the dual consecutive concentrations were prepared with the range of 8192 to 16 μg/mL and transferred to microplate wells. The concentration of inoculated bacteria per well was 10^5^ CFU/mL. The wells without the yeast supernatant and lysate extracts were considered as positive control and the wells free of bacteria as negative control. The microplate was transferred to an incubator at 37°C and the wells were examined for growth after 20 hours. The lowest concentration of yeast supernatant and lysate extracts without turbidity was determined as the MIC value. The colonies were counted in the turbidity-free wells, and the lowest concentration that reduced the bacterial population by 99.9% was recorded as the MBC value. Methanol was the solvent of supernatant extract. To ensure the inactivation of methanol inhibition in the measured concentrations, methanol MIC was also determined by broth microdilution.

### Study of bacterial biofilm formation.

The micro-titer plate assay was used to investigate biofilm formation by *S. aureus* strains (19). The bacteria were cultured in the TSB medium for 24 hours. The cultures were diluted at a ratio of 1: 100, with TSB containing 1% glucose (TSBGlc) to give a suspension of OD_600_ = 0.1 (10^8^ CFU/ml). From this suspension, 200 μL was transferred to microplate wells. Three wells were considered for each bacterial strain. Moreover, the bacteria-free TSB medium was used as a control. The microplate was incubated for 24 hours at 37°C. The medium was drained inside the wells and washed 3 times with the PBS solution. After the wells were dried, the biofilm was first fixed with 95% ethanol and then stained with 100 μL of crystal violet dye 1% for 5 minutes. Next, the wells were washed by tap water to remove extra dye. After wells were dried, 150 μL of acetic acid (33%) was added to the wells so that the crystal violet dye was released and dissolved. The optical density (OD) of wells was read at a wavelength of 490 nm by a microplate reader (ELx808, BioTek, USA). The mean OD of the 3 wells for each strain was recorded as OD_t_, and the 3 wells for control as OD_c_, and the biofilm grade was determined according to Table 1 (20).

**Table 1.** Interpretation of optical density (OD) results read in microplate wells (20)

**OD**	**Result**
OD_t_ ≤ OD_c_	non-biofilm
OD_c_ < OD_t_ < 2× OD_c_	weak biofilm
2× OD_c_ < OD_t_ < 4× OD_c_	moderate biofilm
OD_t_ ≥ 4× OD_c_	strong biofilm

### Effect of the yeast supernatant and lysate extracts on biofilm formation.

At first, the bacterium was cultured in the TSB medium for 24 hours, and then 10^8^ CFU/mL of the bacteria was inoculated into TSBGlc medium containing different concentrations of 512, 1024, and 2048 μg/mL of the yeast supernatant and lysate extracts. From this suspension, 200 μL was transferred to microplate wells. The extract-free well was used as a positive control and the bacteria-free well as a negative control. The microplate was incubated at 37°C for 24 hours, and then biofilm formation was investigated. Finally, the positive control sample was compared with the extracts treated specimens. This experiment was repeated 3 times (21).

### Effect of yeast extracts on hemolytic activity.

The hemolytic activity was measured by the method previously described by Shi et al. with some modifications (22). The bacterium was first cultured in the TSB medium for 24 hours at 37°C. From this culture, 10^8^ CFU/mL was inoculated onto the TSB medium containing different concentrations of 512, 1024, 2048 μg/mL of the yeast supernatant and lysate extracts. The suspension was poured into a flask and incubated at 37°C for 18 hours with 180 rpm. The positive control was an extract-free flask and the negative control was a bacteria-free flask. Next, 1 mL of each flask was poured into the microtube and centrifuged at 7000 rpm to separate the supernatant. To evaluate the hemolytic activity, 100 μL of bacterial supernatant, 875 μL of PBS, and 25 μL of defibrinated rabbit blood were incubated in each microtube for 30 minutes at 37°C. The samples were centrifuged at 7000 rpm for 1 minute at 4°C, and the OD was read at 490 nm. All steps were completed as triplicate.

### Effect of yeast extracts on expression of enterotoxin A and α-hemolysin genes.

At first, the bacterium was cultured in the TSB medium overnight. From this culture, 10^5^ CFU/mL of bacteria was inoculated onto TSB medium containing 2048 μg/mL of yeast supernatant and lysate extracts. The suspension was incubated up to the late log phase at 37°C with 180 rpm. The extract-free culture was used as controls. To prepare cell pellet, the culture was centrifuged at 5000 g for 10 minutes. The cell pellet was kept at −70°C. The cells were lysed by addition of TE buffer containing 40 mg/mL of lysozyme and 100 μg/mL of lysostaphin. Total RNA was extracted using RNeasy Mini kit (Qiagen, Germany), according to manufacturer’s instructions. The concentration and purity of RNA were measured using NanoDrop device (NanoDrop One, Thermo Fisher, USA). QuantiTect Reverse Transcription Kit (Qiagen, Germany) was used to remove genomic DNA contamination and cDNA synthesis. After cDNA synthesis, the real-time PCR process was used to compare the expression levels of *sea* and *hla* genes in supernatant- and lysate-treated samples with the control sample. The sequence of evaluated primers is listed in Table 2. The sequences of the genes were extracted from NCBI GenBank and primers were designed using AlleleID 6 software (*Sea* GenBank: KR296942.1, *hla* GenBank: KT279555.1 and *16s rRNA* GenBank: NR_118997.2). The real-time PCR process was performed in triplicate using Quantitect SYBR Green PCR PCR Kit (Qiagen, Germany) and Rotor-Gene Q (Qiagen, Germany). The Housekeeping 16S rRNA gene was used to normalize the expression of genes. Relative gene expression levels were determined using the ΔΔC_T_ method (23).

**Table 2.** Primers used for quantitative real-time

**Gene**	**Primer Sequence (5′→3′)**	**Product size**
*sea*	F: TATGGTGCTTATTATGGTTATC R: TTTCTCTTCGGTCAATCG	108 bp
*hla*	F: ACATTACCGTTGAATCCATAAG R: ATATAGAGTTTATAGCGAAGAAGG	180 bp
*16s rRNA*	F: TTAGTTGCCATCATTAAGTTG R: CTTTGTATTGTCCATTGTAGC	132 bp

### Statistical analysis.

Concerning the effects of extracts on the biofilm formation and hemolytic activity, the results obtained in the tested specimens were compared with the positive control samples using one-way ANOVA and LSD tests. P value less than 0.05 was considered as statistically significant.

## RESULTS

### MIC and MBC results.

MIC values of the supernatant extract for both strains of *S. aureus* ATCC 29213 (MSSA) and *S. aureus* ATCC 33591 (MRSA) were obtained to be 4096 μg/mL by broth microdilution method. Also, the MBC values were the same as the MIC concentration (4096 μg/mL) for both strains. The lysate extract showed no bacteriostatic and bactericidal effects on any of the 2 standard strains of *S. aureus*. Moreover, methanol had no inhibitory effect on the 2 strains at the measured concentrations.

### Biofilm formatin results.

To evaluate the biofilm produced by the studied strains, the OD values of samples were compared with the control. The results showed strong biofilm formation by both *S. aureus* standard strains (OD_t_ ≥ 4× OD_c _). The OD of the test samples for standard MSSA and MRSA strains were 6.2 and 9.4 times higher than the control, respectively.

### Antibiofilm activity of *S. cerevisiae* extracts.

**Fig. 1.**
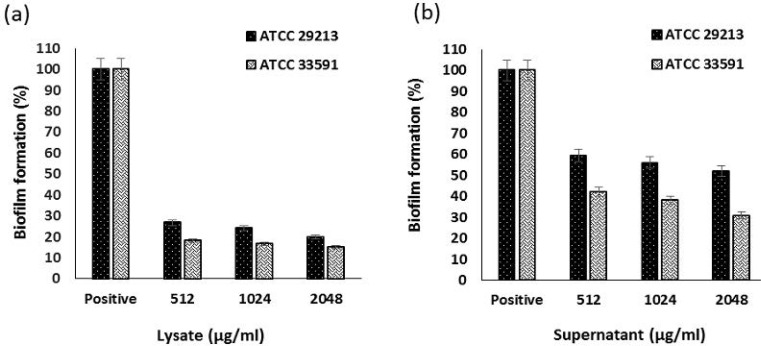
Effect of lysate (a) and supernatant (b) extracts of *S. cerevisiae* S3 on biofilm formation of MSSA and MRSA standard strains

In this study, the effect of 3 sub-MIC concentrations of *S. cerevisiae* S3 supernatant extract on the biofilm formation of *S. aureus* was investigated. These concentrations were 512, 1024, and 2048 μg/mL. The same concentrations were also used to evaluate the antibiofilm effect of lysate extract yeast. Both *S. aureus* strains were strong biofilm producers; and both supernatant and lysate extracts could significantly reduce the biofilm formation of MRSA and MSSA (*P* <0.001) at all concentrations. In the case of lysate extract, this effect was somewhat dependent on concentration in both strains, and increased concentration resulted in a further decrease in biofilm formation, so a significant difference was observed between the concentrations of 2048 μg/mL and 512 μg/mL (*P* <0.05). The effect of supernatant extract on MRSA strain followed a concentration-dependent manner. There was a significant difference (*P* <0.05) between the concentrations of 2048 μg/mL and 512 μg/mL, but no significant difference was observed between these concentrations for MSSA strain. At the highest concentration (2048 μg/ml), the lysate extract reduced the biofilm formation in both strains by more than 80%, which means reducing biofilm formation to a weak degree. Also, the highest concentration of supernatant extract resulted in 48% decrease in biofilm formation in MSSA strain and 69% reduction in MRSA strain, which means a decrease in the biofilm formation of both strains to a moderate degree (Fig. 1).

### Antihemolytic activity of *S. cerevisiae* extracts.

This study examined the hemolytic activity of 2 standard strains of MSSA and MRSA. The MSSA strain showed more hemolytic activity. The comparison of ODs between positive control samples of 2 strains showed that hemolysis level of rabbits’ RBC in exposure to supernatant of MSSA strain was 1.98 times greater than that of MRSA strain. The OD values of positive controls were, respectively, 0.365 for MSSA strain and 0.184 for MRSA strain.

**Fig. 2.**
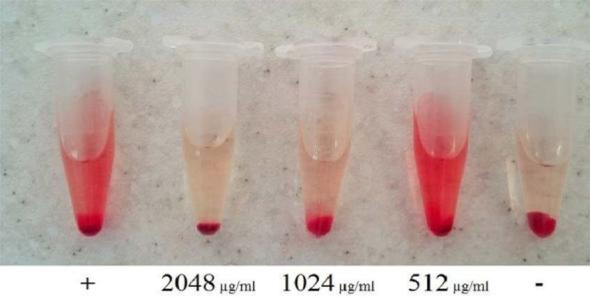
Effect of supernatant extract on hemolytic activity of MRSA standard strain

The supernatant extract was able to significantly reduce the hemolytic activity of MRSA strain at all concentrations (*P* <0.001) (Fig. 2), while only 2 concentrations of 2048 and 1024 μg/mL of supernatant extract caused a significant reduction in the hemolytic activity of MSSA strain. In the case of supernatant extract, this effect was concentration-dependent and the increased concentration caused more reduction in hemolysis, so there was a significant difference between the concentrations (*P* <0.05).

**Fig. 3.**
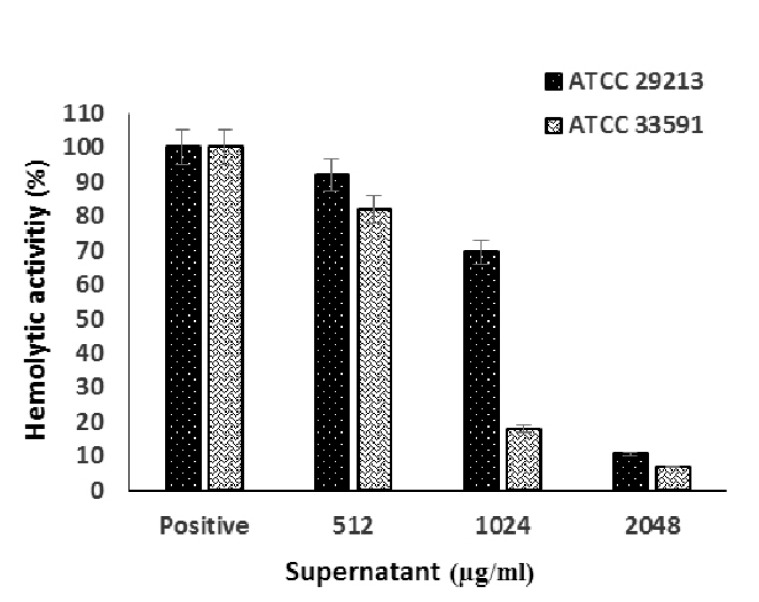
Effect of *S. cerevisiae* S3 supernatant extract on hemolytic activity of MSSA and MRSA standard strains

The supernatant extract at the highest concentration can reduce the hemolytic activity of MSSA and MRSA strains by 90% and 93%, respectively (Fig. 3). However, the lysate extract in none of the concentrations could significantly decrease the hemolytic activity of the 2 *S. aureus* strains (*P* > 0.05).

### Real-time PCR results.

**Fig. 4.**
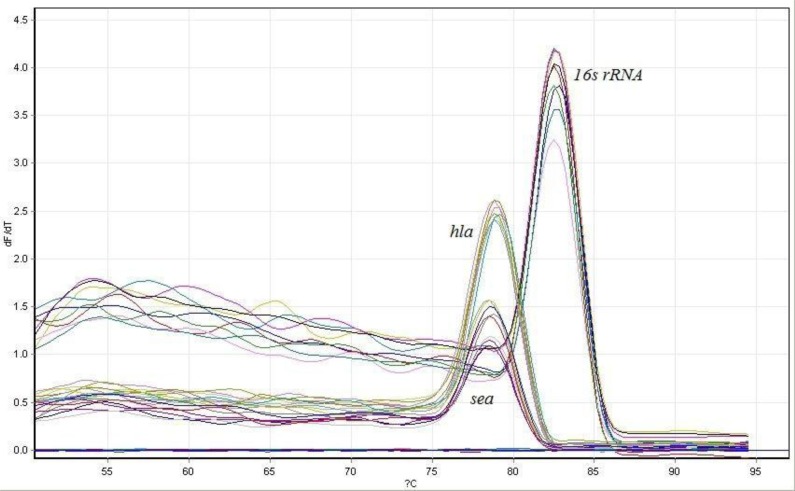
Real-time PCR melting curve

The effect of supernatant and lysate extracts of *S. cerevisiae* S3 on the expression level of *hla* and *sea* genes in the 2 standard strains of MSSA and MRSA were evaluated using real-time PCR. First, the real-time PCR process melting curve was explored to ensure the absence of contamination or primer dimer. The presence of one peak for each gene on the melting curve indicated the correctness of the reaction (Fig. 4).

The results showed that the *sea* gene expression in MRSA strain was 1.84 times greater than that of MSSA strain. On the other hand, the *hla* gene expression in the MSSA strain was 1.5 times higher than that of MRSA strain. In other words, the MRSA strain produced more enterotoxins A and less α-hemolysin than MSSA strain.

**Fig. 5.**
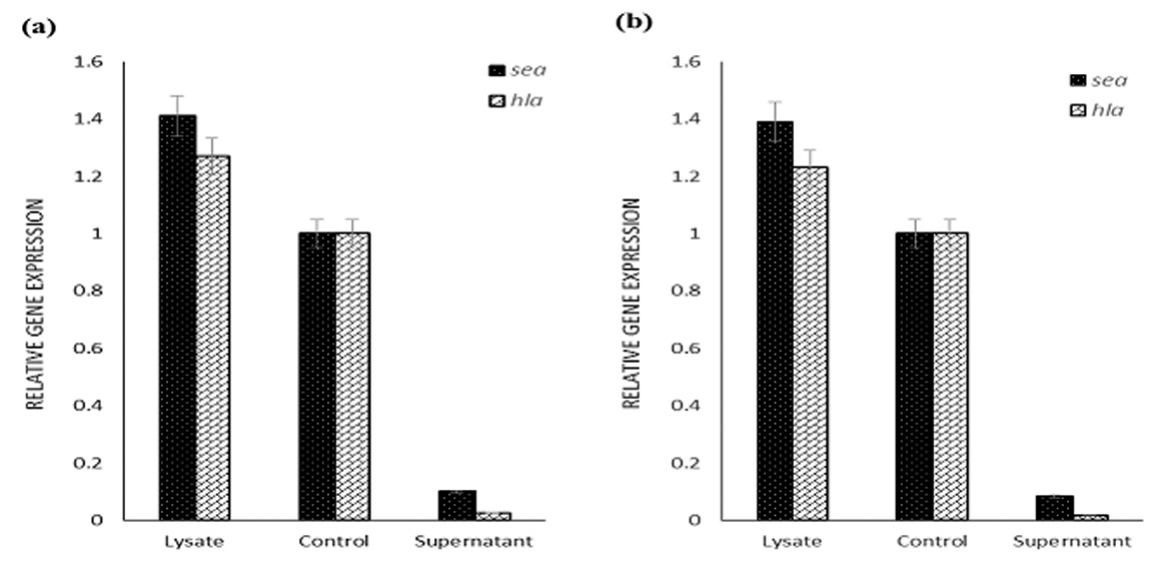
Effect of supernatant and lysate extracts of *S. cerevisiae* S3 on the expression of *sea* and *hla* genes in MSSA (a) and MRSA (b) standard strains

The yeast supernatant extract could reduce the expression of the *sea* gene in both *S. aureus* strains. This extract caused a 10-fold reduction of the *sea* gene expression in MSSA strain and a 12-fold decrease in MRSA strain. The supernatant extract in both *S. aureus* strains reduced *hla* gene expression. The *hla* gene expression was decreased by 40 folds in the MSSA strain and by 71 folds in MRSA strain. The lysate extract had no inhibitory effect on the expression of *sea* and *hla* genes and increased the expression of these genes in both *S. aureus* strains (Fig. 5).

## DISCUSSION

*S. aureus* is one of the most important causes of nosocomial infection (eg, indwelling medical device-associated infections and food poisoning) (22). The bacterial resistance to antibiotics is among the greatest challenges threatening the health of the modern humans (24). The use of natural compounds produced by other microorganisms, such as probiotics, can be a solution to prevent and control the infections (16). One of the important roles of probiotic yeasts has been reported to be antagonistic properties against other microorganisms (13). Most studies on antibacterial activity of yeasts have used *S. boulardii*, but several new studies used *S. cerevisiae.* Srinivas et al. examined the antagonistic activity of *S. cerevisiae* OBS2 against several bacterial pathogens including *S. aureus* using agar well diffusion test. The results showed antimicrobial activity of this strain against *S. aureus* and other pathogens (25). Lima et al examined the antagonistic activity of 28 strains of *S. cerevisiae* against different pathogenic bacteria. Among strains tested, 15 strains had shown antimicrobial activity against *S. aureus* (26). In a study conducted by Fakruddin et al., the antagonistic activity of supernatant and lysate of yeast *S. cerevisiae* IFST062013 was investigated against different pathogens using agar well diffusion method. However, the supernatant and lysate extracts were not prepared. The results showed that both supernatant and lysate had antimicrobial properties against *S. aureus* (12). The present study was conducted to investigate the effect of supernatant and lysate extracts of probiotic yeast *S. cerevisiae* S3 on *S. aureus*. In this study, only the supernatant extract had antibacterial activity and the lysate extract had no effect. This may be due to the degradation of some of the lysate metabolites during drying or may be due to the use of different *S. cerevisiae* strains from those used in the study of Fakruddin et al. (12).

The performance of antimicrobial agents to treat the infections is both dependent on their bactericidal and bacteriostatic effects and on their ability to inhibit the production of bacterial virulence factors (10). *S. aureus* has a number of virulence factors that play an important role in pathogenesis. This study examined the effects of supernatant and lysate extracts of *S. cerevisiae* S3 on 3 virulence factors of *S. aureus*, including biofilm, α-hemolysin and enterotoxin A. In the present study, both supernatant and lysate extracts showed a significant decrease in biofilm formation in both standard MSSA and MRSA strains. Although the lysate extract has shown no bacteriostatic and bactericidal effects, it was able to reduce biofilm formation more effectively. Moreover, this study revealed that the biofilm formation in the MRSA strain was reduced further by the effect of both extracts. Previous studies have shown that biofilm formed by MSSA and MRSA strains is phenotypically different, which may have been the cause of the difference in the level of effect of extracts. The MSSA strains generally produce PIA-dependent biofilm, while the biofilm formation in MRSA strains is predominantly independent of PIA and is due to surface binding protein and extracellular DNA (eDNA) (27). The only study found on the effect of *S. cerevisiae* on biofilm formation of staphylococci was a study by Walencka et al. (28). In their research, mannoproteins extracted from the cell wall of *S. cerevisiae* decreased the biofilm growth and facilitated the disperse of cells from mature biofilm. Several studies have been conducted on the effects of other probiotics on the biofilm formation of *S. aureus*, which showed reduced formation or degradation of biofilm. Melo et al. demonstrated that the sub-MIC value of *Lactobacillus fermentum* supernatant can significantly reduce the biofilm formation of *S. aureus* (29). Merghni et al., investigated the anti-biofilm effects of biosurfactant derived from *L. fermentum* on oral strains of *S. aureus* and their results revealed reduced biofilm formation and degradation of the created biofilms (30).

The authors of this study did not find any published report on the probiotic yeast effects on virulence gene expression of *S. aureus* on validated databases. Thus, the results of this study could not be compared with those of similar studies. However, similar studies have been found on probiotic lactobacilli. Even et al. investigated the coculture effect of *Lactobacillus lactis* on the expression of *S. aureus* virulence factors. It was observed that the expression of several regulators, including *agr* and *sarA* loci, as well as virulence factors, such as enterotoxins, were strongly influenced (31). Parsaeimehr et al. showed that the mixed culture of *Lactobacillus acidophilus* and *Lactobacillus casei* reduced the *sea* gene expression in *S. aureus* (32). In the present study, the *S. cerevisiae* S3 lysate extract showed no inhibitory effect on the expression of *sea* and *hla* genes in *S. aureus*, but the supernatant extract of this yeast could reduce the expression of both genes. The difference between the effects of supernatant and lysate extracts may be due to their different compounds, which requires further studies to identify them. The results of α-hemolysin gene expression were similar to those of the hemolytic activity; the lysate extract in the phenotypic method did not reduce hemolysis, but the supernatant extract was able to significantly reduce hemolysis, and this decrease was higher in the MRSA strain. As demonstrated in the results, the MSSA strain had more hemolytic activity than the MRSA strain (1.98 fold), and the expression of the *hla* gene was also higher in this strain (1.5 fold). The difference in the rate of supernatant extract’s effect on the hemolytic activity of the 2 strains may be due to this reason. Therefore, the results promisingly suggest the use of compounds produced by the probiotic yeast *S. cerevisiae* S3 in controlling and preventing *S. aureus* infections.

## CONCLUSION

The present study demonstrated the favorable antagonistic effects of *S. cerevisiae* on the methicillin-resistant and sensitive strains of *S. aureus*. According to the results of this study, the compounds produced by probiotic yeast *S. cerevisiae* can be used to inhibit the growth and production of *S. aureus* virulence factors. Also, conducting further studies on this topic is highly recommended.
